# Treating Caregiver Grief With Narrative Therapy

**DOI:** 10.1093/geroni/igab046.3750

**Published:** 2021-12-17

**Authors:** Tara Matta

**Affiliations:** University of South Florida, Tampa, Florida, United States

## Abstract

Dementia, a devastating neurodegenerative disease with over 10 million new diagnoses each year, is characterized by many symptoms including memory loss .Individuals with memory less experience changes in mood, personality, behavior, cognition and activities of daily living which affect their daily lives. These monumental life shifts often occur rapidly, leaving caregivers unprepared to deal with the changes. Caregivers face a unique situations navigating anticipatory grief and changes in their relationships with their loved ones. Current psychological intervention for caregivers includes utilization of cognitive-behavioral therapy and psychoeducation. More recently, intriguing research has emerged regarding the efficacy of narrative therapy for couples where one partner experiences memory loss. However, treating the anticipatory grief component specifically for caregivers has been largely overlooked in these studies. Narrative therapy revolves around identifying the current story that caregivers utilize as their cognitive framework, helping to find alternative plotlines and to process their newly-built cognitive framework. It involves externalizing the problem (in this case, dementia) and locating strengths that the caregiver and their care receiver share to “fight” the problem. Insights from both the current literature and the field have demonstrated a promising outlook on the use of narrative therapy. Such insights imply a need for more research regarding this modality specifically for caregivers, as its core ideas can be easily disseminated to gerontologists, mental health professionals and caregivers.

